# Supplementary material for: Quantitating the epigenetic transformation contributing to cholesterol homeostasis using Gaussian process
Source: Nat Commun. 2019 Nov 7;10:5052. doi: 10.1038/s41467-019-12969-x (PMC6838179; doi:10.1038/s41467-019-12969-x)
Supplement: Supplementary file 1 — Supplementary Information [file 41467_2019_12969_MOESM1_ESM.pdf]

Supplementary Information

Quantitating the Epigenetic Transformation Contributing to Cholesterol Homeostasis Using Gaussian Process

Chao Wang[1], Samantha M. Scott[1], Kanagaraj Subramanian[1], Salvatore Loguercio[1], Pei Zhao[1], Darren Hutt[1], Nicole Y. Farhat[2], Forbes D. Porter[2], and William E. Balch[1*]

[1]Department of Molecular Medicine, Scripps Research, La Jolla, CA, 92037, USA.[2] Section on Molecular Dysmorphology, Division of Translational Medicine, *Eunice Kennedy Shriver* National Institute of Child Health and Human Development, National Institutes of Health, Bethesda, MD, 20814, USA.

*Address correspondence to: webalch@scripps.edu

Supplementary Table 1

Human NPC1 patient fibroblasts used in this study

GM5659	Wild type
GM17920	P401T/I1061T
GM17913	V1165M/I1061T
GM17912	P1007A/T1036M
GM18453	I1061T/I1061T
GM18436	E612D/F542fsX
GM18398	G673V/I1061T

Supplementary Table 2

Classification of NPC1 variants according to trafficking index (TrIdx) in HeLa cells

**Class I**	**Class II**	**Class III**	**Class IV**
No synthesis	ER	ER-------E/L	E/L
-	0-20%	20-50%	>50%
G655X	C63R→	P543L	L80V
S738X	R389L	Y634C	C177Y
	R404Q→	S666N→	C227W
	H510P→	P691S	P237S
	R518Q	Y825C	H215R
	P532L→	I858V	D242H
	E612D→	D874V	V378A←
	R615L→	S954L→	C479Y
	S636F→	R958Q	I642M
	G673V→	G992R	L648H←
	P887L	P1007A→	S734I
	Q921P	T1036M	V1212L
	I923V	A1054T	M1142T
	R934Q→	R1077Q→	
	S940L→→	Y1088C	
	W942C	W1145R	
	D948N→	G1162A	
	V950M→→	V1165M	
	R1059Q	R1186G	
	I1061T	A1187V	
	A1151T→	L1191F	
	3565-66 insG		
	G1240R→		
	L1244P→		
	3741-44del		

→ Variant is classified as one class higher category based on TrIdx value in U2OS cells.

→→ Variant is classified as two classes higher category based on TrIdx value in U2OS cells.

← Variant is classified as one class lower category based on TrIdx value in U2OS cells.

Supplementary Table 3

Natural history information of patients included in this study

Patient code	Allele 1 variation	Allele 2 variation	Severity Score	Age of first neurologic symptom	Allele 1 TrIdx Class	Allele 2 TrIdx Class
NPC1	I1061T	I1061T	35	9	2	2
NPC2	V1165M	fs3741-44del (L1247fs)	5	5	3	2
NPC3	V1165M	fs3741-44del (L1247fs)	33	3	3	2
NPC4	I1061T	I1061T	11	3	2	2
NPC5	I1061T	R1186G	14	3	2	3
NPC6	I1061T	P237S	18	3	2	4
NPC9	I1061T	M1142T	7	2	2	4
NPC16	P887L	fs3741-44del(L1247fs)	2	5.7	2	2
NPC24	I1061T	I1061T	35	5	2	2
NPC27	I1061T	I1061T	4	3.4	2	2
NPC36	I1061T	R404Q	27	1.5	2	2
NPC37	S734I	S734I	24	5	4	4
NPC41	I1061T	T1036M	4	3.6	2	3
NPC44	I1061T	P1007A	14	13	2	3
NPC46	I1061T	P543L	0	2	2	3
NPC47	I1061T	I1061T	13	3.5	2	2
NPC48	R404Q	S954L	21	11	2	3
NPC49	C177Y	V950M	2	7.5	4	3
NPC50	I1061T	P1007A	17	8	2	3
NPC52	I1061T	I1061T	12	1	2	2
NPC53	I1061T	S954L	20	18	2	3
NPC60	R404Q	P1007A	33	6	2	3
NPC63	I1061T	D948N	2	2	2	2
NPC67	T1036M	S954L	20	9	3	3
NPC68	T1036M	S954L	14	8	3	3
NPC69	I1061T	C227W	0	0.8	2	4
NPC70	C479Y	S940L	11	0.5	4	2

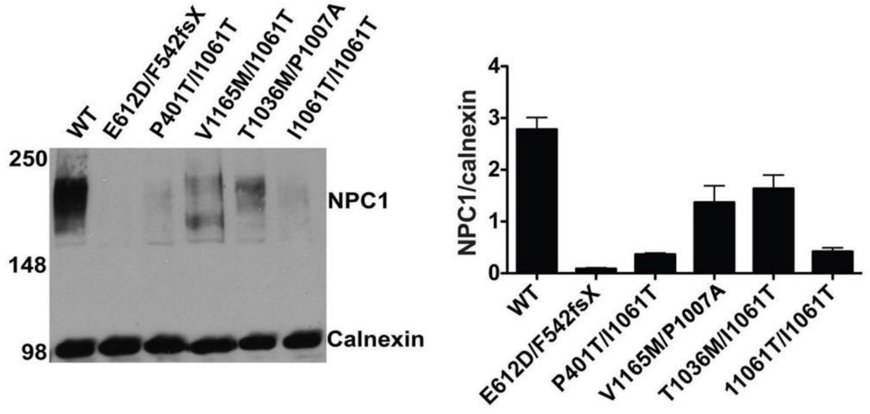

Supplementary Fig. 1. The endogenous level of NPC1 protein found in patient fibroblasts. Cell lysates from patient fibroblasts (Supplementary Table 1) were subjected to SDS-PAGE and Western blotting with rabbit anti-NPC1 antibody. Calnexin was used as loading control.

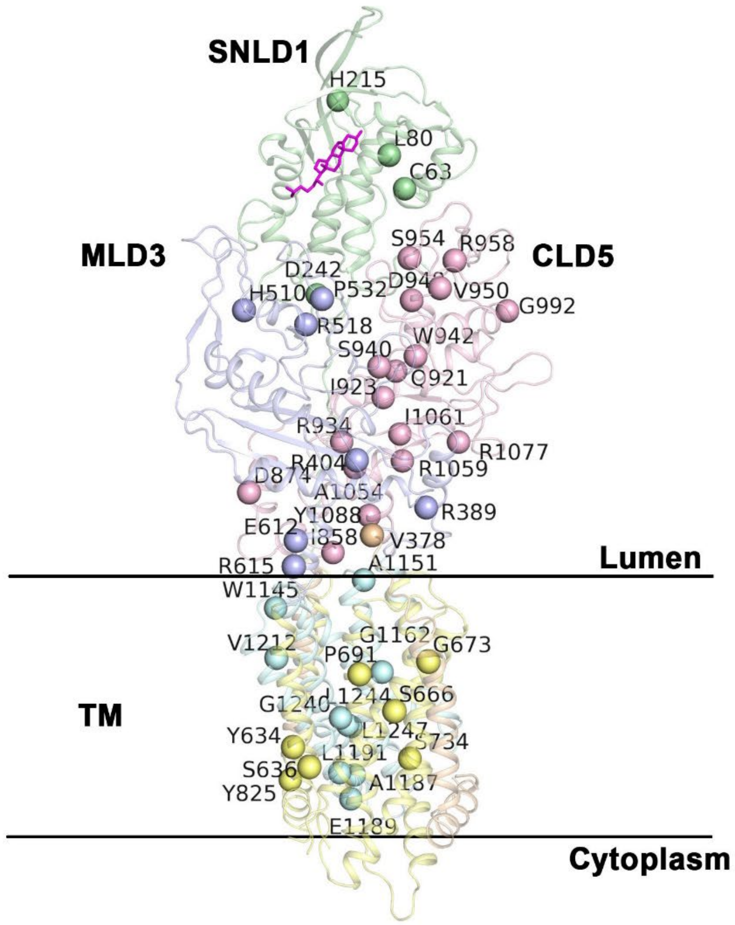

Supplementary Fig. 2. Location of NPC1 variants on the NPC1 structure. NPC1 variants mapped as balls on the C-alpha position of the NPC1 structure (PDB:3JD8 and PDB: 5U73)[1,2]. The variant residue positions in the NPC1 sequence used in the current study are labeled.

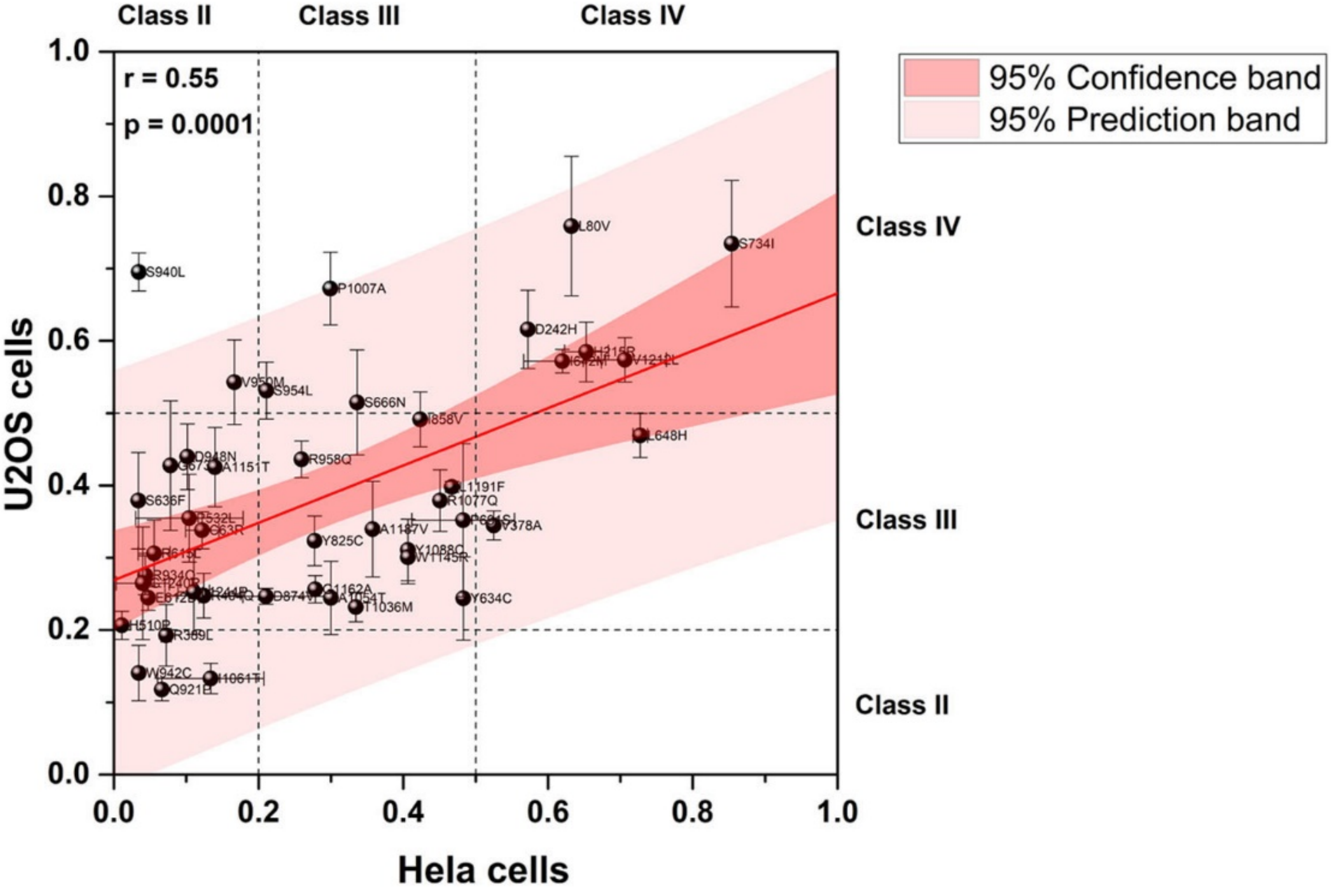

Supplementary Fig. 3. Correlation between trafficking measurements in Hela and U2OS cells. Pearson's r-value and the p-value (ANOVA test) with null hypothesis as the coefficient equal to zero are indicated. The TrIdx class II, III and IV are highlighted by dash lines. Data are shown in mean ± s.d. (Source data are provided as a Source Data file). The variants in class III and class IV are mostly consistent in the two cell lines. In contrast, many class II variants measured in Hela cells move to class III in U2OS cells, which may reflect the cell-specific trafficking environment for those variants or reflect the conformational preference of antibody since NPC1 is immunoprecipitated (IP) by an NPC1 specific antibody for Endo H digestion and Western blotting from Hela cell extracts, while whole cell lysates are used for Endo H digestion and Western blotting using U2OS cells (see Methods).

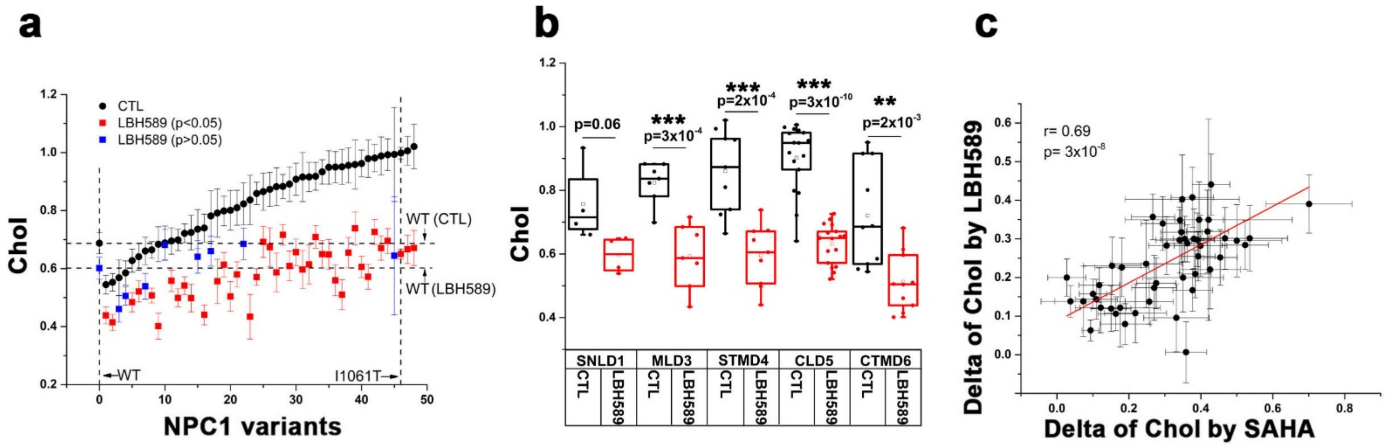

Supplementary Fig. 4. Impact of LBH589 on NPC1 variant Chol homeostasis. (**a**) Chol measurement of NPC1 variants in response to LBH589 [3]. 48 NPC1 variants that were tested for their response to 50 nM LBH589. Response of each variant plotted based on the LE/LY filipin staining value (Chol) in the absence (CTL, black circles) or presence of LBH589 (red and blue squares). Red squares show variants where the LBH589 impact is significant (Student's two-tailed t-test, p-value <0.05). Blue squares show variants where the LBH589 effect is not significant (Student's two-tailed t-test, p-value >0.05). WT and I1061T variants are indicated by vertical dash lines and the Chol value of WT in the absence (CTL) or presence of LBH589 are highlighted by horizontal dash lines. Data is shown as mean ± s.e.m (Source data are provided as a Source Data file). (**b**) Domain specific response to LBH589. NPC1 variants are clustered into different domains shown in box and whisker plots (box = 25th and 75th percentile; whisker length extend from the minimum to maximum of the data; p-value (Student's two tailed t-test)) in the absence (CTL, black boxes) and presence (red boxes) of LBH589. (**c**) Correlation between the effect of SAHA and LBH589 on the Chol homeostasis. Data is shown as mean ± s.e.m.[3] Pearson's r-value and the p-value (ANOVA test) with null hypothesis as the coefficient equal to zero are indicated.

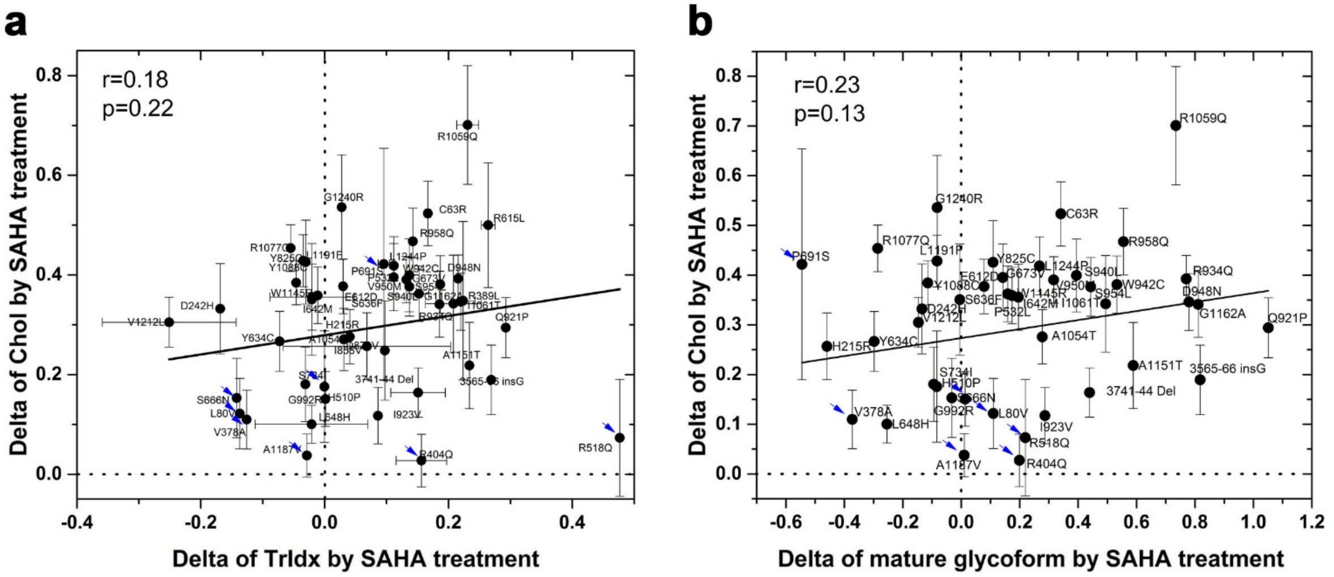

Supplementary Fig. 5. Correlation between trafficking response and Chol response to SAHA. Correlation between the delta (Δ) of Chol to delta (Δ) of TrIdx (**a**) or delta (Δ) of absolute mature glycoform (Endo H^R) (**b**) in response to SAHA treatment is presented. Pearson's r-value and the p-value (ANOVA test) with null hypothesis as the coefficient equal to zero are indicated. Variants that are not significantly corrected by SAHA for cholesterol homeostasis are indicated by blue arrows. Data is shown as mean ± s.e.m.

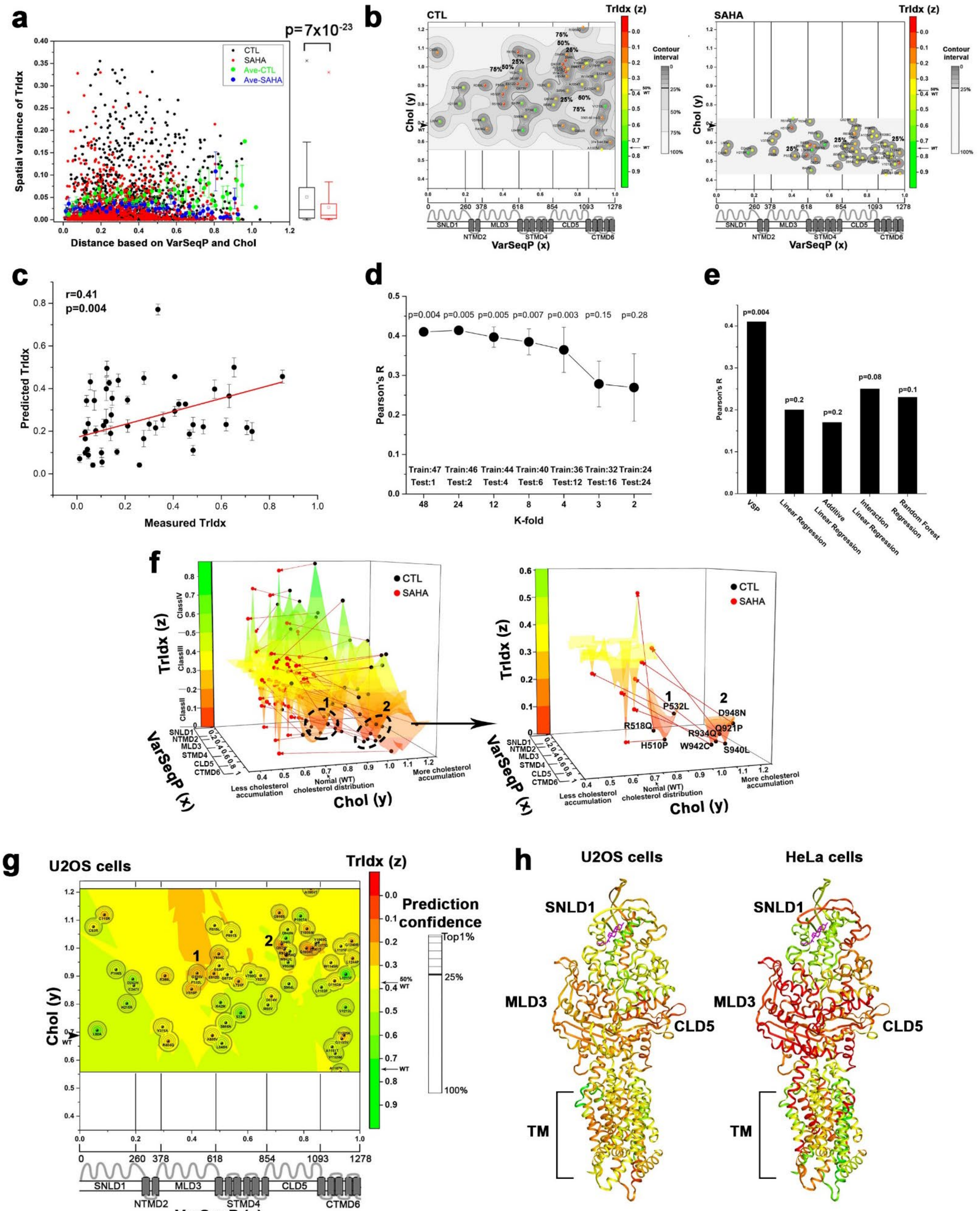

Supplementary Fig. 6. VSP analysis of TrIdx-phenotype landscapes. (**a**) The spatial variance of TrIdx and the distance based on VarSeqP and Chol for all possible 1128 variant pairwise comparison (Fig. 2a, black lines)

were calculated and plotted with black circles representing the spatial variance in the vehicle control condition (DMSO) and red circles representing the spatial variance in the SAHA condition. The comparison of spatial variance between control (black) and SAHA (red) is shown as a box and whisker plot at right margin (box = 25th and 75th, whisker length = outmost data point in the inner fence; p-value (Student's two tailed t-test)). The spatial variance defined by the black and red circles are binned by the distance interval of 0.02. The average (mean ± s.e.m) of spatial variance is calculated and shown as green circles (control) or blue circles (SAHA) (see Methods). (**b**) Confidence in SCV relationships seen in the TrIdx-phenotype landscape in the absence (left panel) and presence (right panel) of SAHA within the variogram range is plotted as a gray gradient delineated by contour lines in the 2D map. The top 25% confidence quartile is shown as a bold line. The SCV relationships in the top 25% confidence region are of high confidence and spatially dependent on one another from both trafficking and conductance perspectives. SCV relationships outside the top 25% confidence quartile (bold line) progressively approach (control, left) or arrives at (SAHA, right) the plateau value in the molecular variogram and are therefore of lower confidence in assessing SCV relationships (Fig. 2b in the main text). (**c**) Cross-validation result for the TrIdx-phenotype landscape. A leave-one-out cross-validation analysis demonstrates a significant correlation between all measured and predicted values in the output TrIdx-phenotype landscape. Pearson's r-value and the p-value (ANOVA test) with null hypothesis as the coefficient equal to zero are indicated. Error bars represent the variance associated with each prediction (see Methods). (**d**) *k*-fold (see Methods) cross-validation result shows that prediction accuracy is significant until the number of training datapoints fall below ~36. (**e**) Comparison of the leave-one-out validation result of VSP to other regression methods including simple linear regression of TrIdx and Chol relationships, as well as multivariate linear regression models (e.g., additive linear regression and interaction linear regression) and decision-tree method (Random Forest regression) using the same datasets as VSP (i.e., VarSeqP, TrIdx and Chol). VSP achieves the best cross-validation result when compared with other methods, and importantly, regression methods other than Gaussian process do not explicitly assess the uncertainty/confidence of the prediction which is important to link sequence-to-function-to-structure. (**f**) (Left panel) 3D projection of TrIdx-phenotype landscape (Fig. 2c in main text) for each variant in the absence (black circles) or presence (red circles) of SAHA. Trajectory of correction indicated by red arrows. SCV clusters 1 and 2 are selectively highlighted in the right panel. (**g-h**) TrIdx-phenotype landscape (**g**) and TrIdx-functional structure (**h**, left panel) built on TrIdx measurements from U2OS cells. When compared to the TrIdx-functional structure in Hela cells (**h**, right panel), even though the red regions observed HeLa cells (reflecting a high trafficking impact), are reduced in U2OS cells (**h**, left panel, yellow to orange), the two TrIdx-functional structures have similar patterns with MLD3 and CLD5 handshake dominating the trafficking phenotype of NPC1.

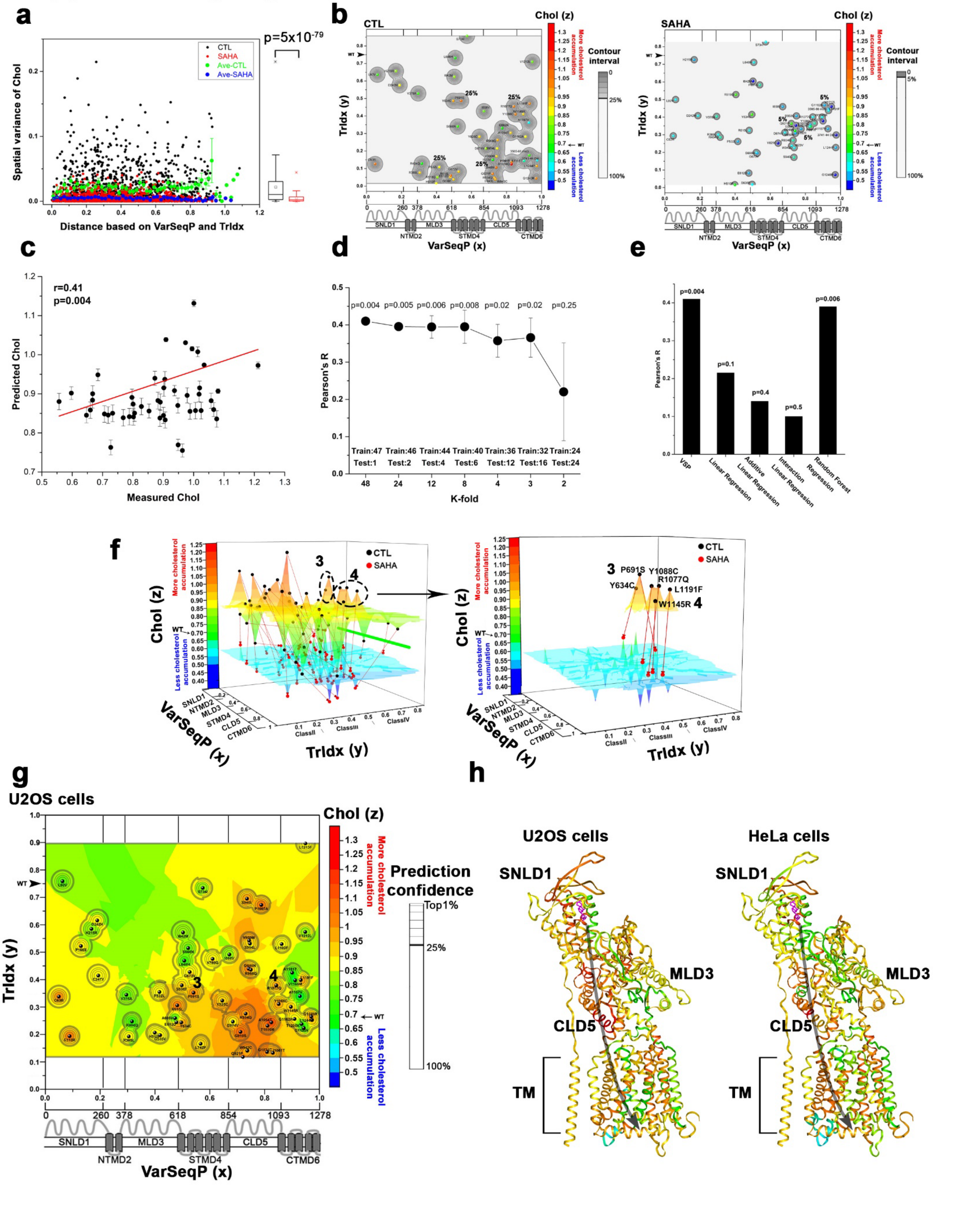

Supplementary Fig. 7. VSP analysis of Chol-phenotype landscapes. (**a**) The spatial variance of Chol and the distance based on VarSeqP and TrIdx for all possible 1128 variant pairwise comparison (Fig. 3a, black lines)

were calculated and plotted with black circles representing the spatial variance in vehicle control condition (DMSO) and red circles representing the spatial variance in the presence of SAHA. The comparison of spatial variance between control (black) and SAHA (red) is shown as box and whisker plot at right margin (box = 25th and 75th, whisker length = outmost data point in the inner fence; p-value (Student's two tailed t-test)). The p-value (Student's two tailed t-test) is indicated. The spatial variance clouds (the black and red circles) are then binned by the distance interval of 0.02. The average (mean ± s.e.m) of spatial variance is calculated and shown as green circles (control) or blue circles (SAHA). (**b**) Confidence in SCV relationships seen in the Chol-phenotype landscape in the absence (left panel) and presence (right panel) of SAHA within the variogram range is plotted as a gray gradient delineated by contour lines in a 2D map. In the control condition, the top 25% confidence contour interval is the variogram range and shown as a bold line. In the SAHA condition, the top 5% confidence contour is the variogram range and shown as a bold line. (**c**) Cross-validation result for Chol-phenotype landscape. A leave-one-out cross-validation analysis demonstrates a significant correlation between all measured and predicted values in the output Chol-phenotype landscape. Pearson's r-value and the p-value (ANOVA test) with null hypothesis as the coefficient equal to zero are indicated. Error bars represent the variance associated with each prediction (see Methods). (**d**) *k*-fold (see Methods) cross-validation result shows that prediction accuracy is significant until the number of training datapoints fall below ~32. (**e**) Comparison of the leave-one-out validation result of VSP to other regression methods including simple linear regression of TrIdx and Chol relationships, as well as multivariate linear regression models (e.g., additive linear regression and interaction linear regression) and decision-tree method (Random Forest regression) using the same datasets as VSP (i.e., VarSeqP, TrIdx and Chol). VSP achieves the best cross-validation result when compared with other methods, and importantly, regression methods other than Gaussian process do not explicitly assess the uncertainty/confidence of the prediction which is important to link sequence-to-function-to-structure. (**f**) 3D Chol-phenotype landscapes (Fig. 3c in main text) showing the impact of SAHA on correction of Chol homeostasis across the entire NPC1 polypeptide chain. The response of each variant to SAHA is shown as red arrows linking variants in the absence (black circles) or presence (red circles) of SAHA. The specific response of SCV clusters 3 and 4 are selectively highlighted in the right panel. (**g-h**) Chol-phenotype landscape (**g**) and Chol-functional structure (**h**, left panel). When compared to the Chol-functional structure built on TrIdx in Hela cells (**h**, right panel), the Chol-functional structure built on TrIdx in U2OS cells (**h**, left panel) shows similar regions that are critical for cholesterol transfer, indicating the ability of VSP to bridge data from different cell lines.

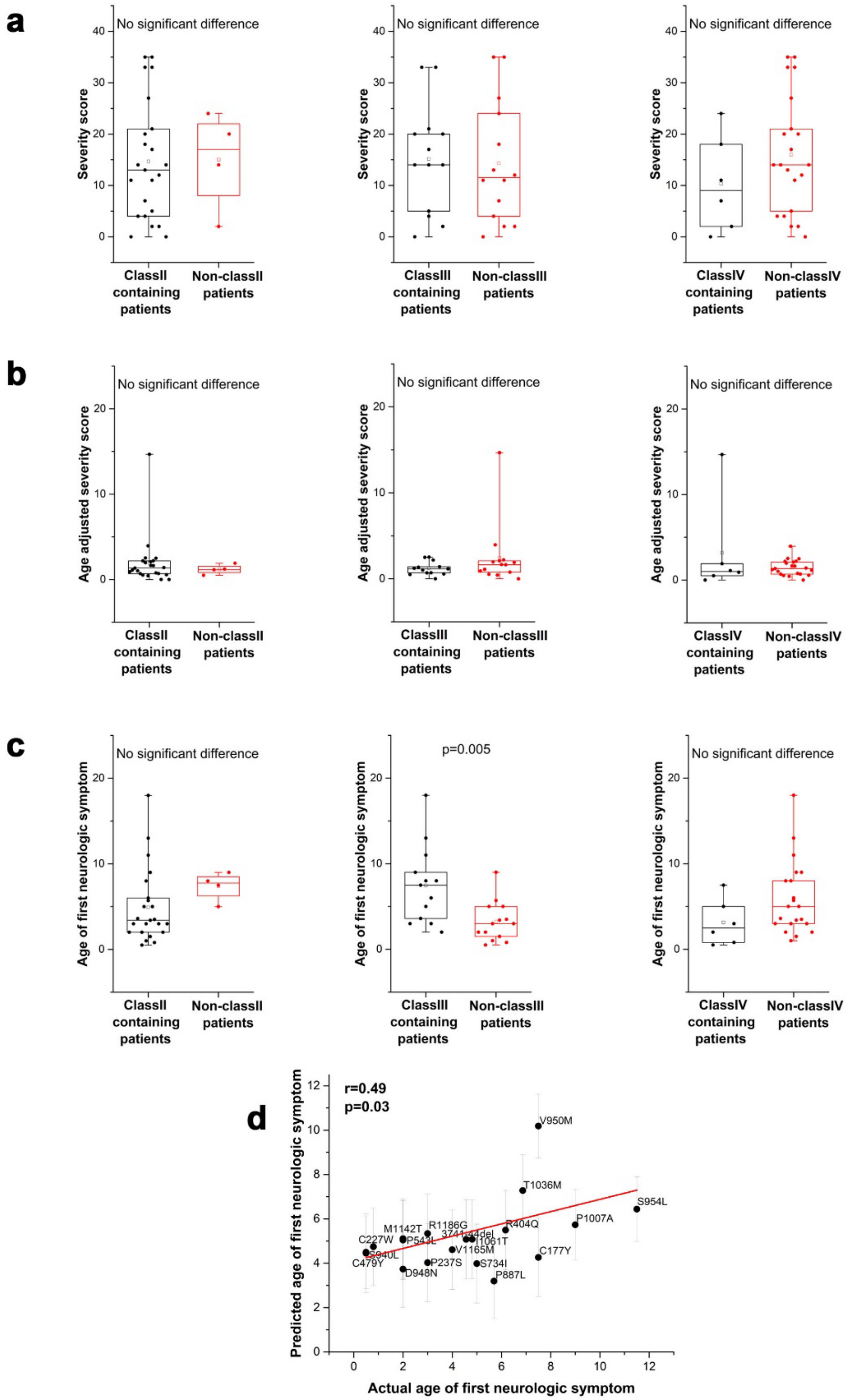

Supplementary Fig. 8. Correlate TrIdx with patient natural history. 27 patients who have variants in both alleles were characterized by the Endo H digestion and Western blotting and are binned as class II containing patients *VS* non-class II patients, class III containing patients *VS* non-class III patients, and class IV containing patients *VS* non-class IV patients. The neurological severity score (see Methods) (**a**), the age adjusted severity score (**b**) and the ANO (**c**) are compared. p-value is calculated by Student's two tailed t-test. Box and whisker plot: box = 25th and 75th, whiskers extend from minimum to maximum of the data. (**d**) Cross-validation result for the ANO-phenotype landscape. A leave-one-out cross-validation analysis demonstrates a significant correlation between all measured and predicted values in the ANO-phenotype landscape. Pearson's r-value and the p-value (ANOVA test) with null hypothesis as the coefficient equal to zero are indicated. Error bars represent the variance associated with each prediction (see Methods).

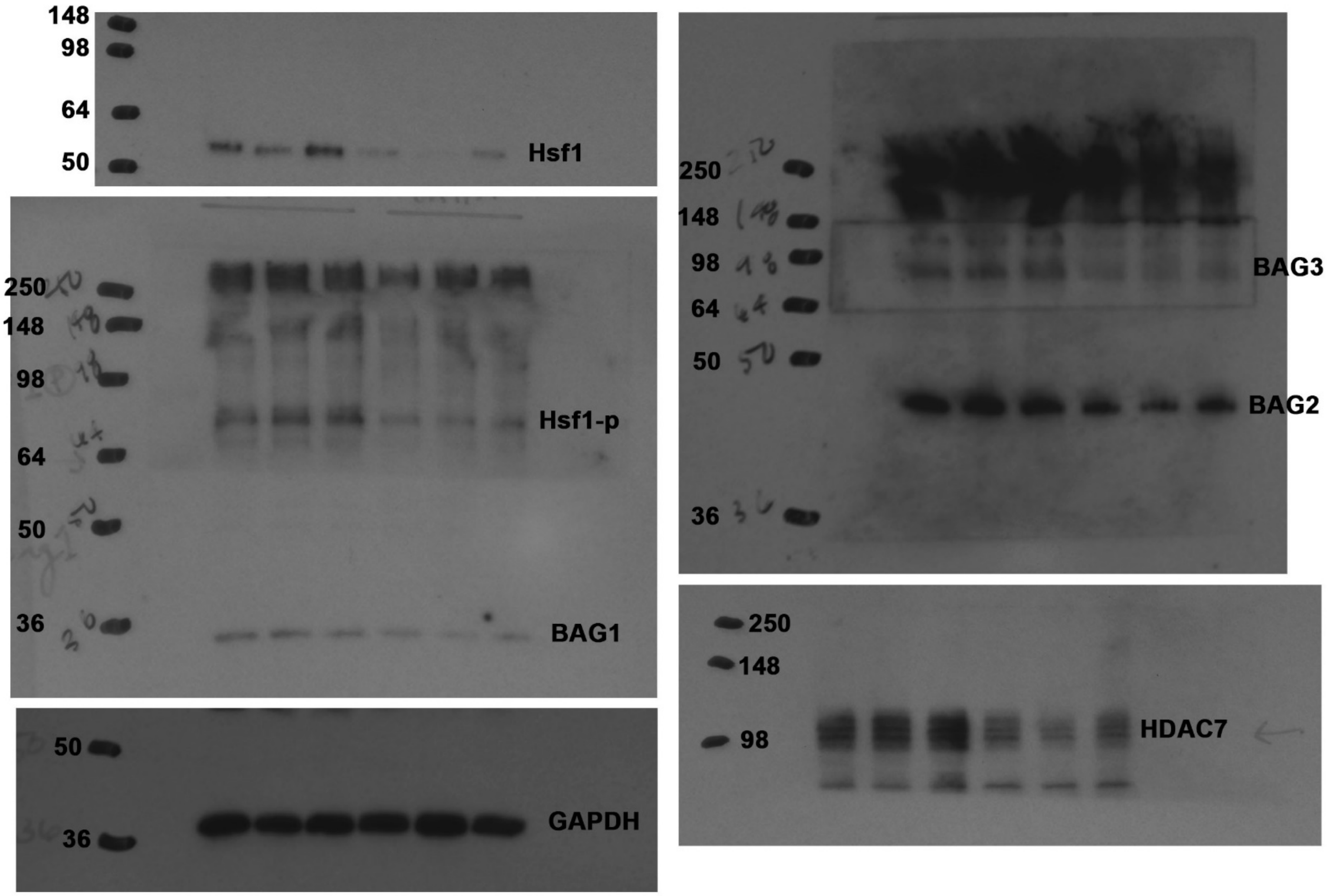

Supplementary Fig. 9. Uncropped blots for Fig. 5b.

Supplementary References

1. Gong, X. *et al.* Structural Insights into the Niemann-Pick C1 (NPC1)-Mediated Cholesterol Transfer and Ebola Infection. *Cell* **165**, 1467-78 (2016).
2. Li, X. *et al.* 3.3 A structure of Niemann-Pick C1 protein reveals insights into the function of the C-terminal luminal domain in cholesterol transport. *Proc Natl Acad Sci U S A* **114**, 9116-9121 (2017).
3. Pipalia, N.H. *et al.* Histone deacetylase inhibitors correct the cholesterol storage defect in most Niemann-Pick C1 mutant cells. *J Lipid Res* **58**, 695-708 (2017).
