# Supplementary material for: Quantitating the epigenetic transformation contributing to cholesterol homeostasis using Gaussian process
Source: Nat Commun. 2019 Nov 7;10:5052. doi: 10.1038/s41467-019-12969-x (PMC6838179; doi:10.1038/s41467-019-12969-x)
Supplement: Supplementary file 3 — Reporting Summary [file 41467_2019_12969_MOESM3_ESM.pdf]

nature research

Corresponding author(s): William E. Balch

Last updated by author(s): Sep 27, 2019

Reporting Summary

Nature Research wishes to improve the reproducibility of the work that we publish. This form provides structure for consistency and transparency in reporting. For further information on Nature Research policies, see Authors & Referees and the Editorial Policy Checklist.

Statistics

For all statistical analyses, confirm that the following items are present in the figure legend, table legend, main text, or Methods section.

n/a	Confirmed	
☐	☒	The exact sample size (*n*) for each experimental group/condition, given as a discrete number and unit of measurement
☐	☒	A statement on whether measurements were taken from distinct samples or whether the same sample was measured repeatedly
☐	☒	The statistical test(s) used AND whether they are one- or two-sided<br>*Only common tests should be described solely by name; describe more complex techniques in the Methods section.*
☐	☒	A description of all covariates tested
☐	☒	A description of any assumptions or corrections, such as tests of normality and adjustment for multiple comparisons
☐	☒	A full description of the statistical parameters including central tendency (e.g. means) or other basic estimates (e.g. regression coefficient) AND variation (e.g. standard deviation) or associated estimates of uncertainty (e.g. confidence intervals)
☐	☒	For null hypothesis testing, the test statistic (e.g. *F*, *t*, *r*) with confidence intervals, effect sizes, degrees of freedom and *P* value noted<br>*Give P values as exact values whenever suitable.*
☒	☐	For Bayesian analysis, information on the choice of priors and Markov chain Monte Carlo settings
☐	☒	For hierarchical and complex designs, identification of the appropriate level for tests and full reporting of outcomes
☐	☒	Estimates of effect sizes (e.g. Cohen's *d*, Pearson's *r*), indicating how they were calculated

Our web collection on statistics for biologists contains articles on many of the points above.

Software and code

Policy information about availability of computer code

Data collection	Image J bundled with Java 1.8.0_112 (NIH image)
Data analysis	GS+, Version 10 (Gammadesign software); Gstat, 1.1-6 (R-package); Originpro 2016 (Originlab); Pymol 1.8.6.0 (Schrodinger, LLC)

For manuscripts utilizing custom algorithms or software that are central to the research but not yet described in published literature, software must be made available to editors/reviewers. We strongly encourage code deposition in a community repository (e.g. GitHub). See the Nature Research guidelines for submitting code & software for further information.

Data

Policy information about availability of data

All manuscripts must include a data availability statement. This statement should provide the following information, where applicable:

- Accession codes, unique identifiers, or web links for publicly available datasets
- A list of figures that have associated raw data
- A description of any restrictions on data availability

All the input and output data were uploaded. See Data availability statement in the manuscript.

Field-specific reporting

Please select the one below that is the best fit for your research. If you are not sure, read the appropriate sections before making your selection.

☒ Life sciences ☐ Behavioural & social sciences ☐ Ecological, evolutionary & environmental sciences

For a reference copy of the document with all sections, see nature.com/documents/nr-reporting-summary-flat.pdf

Life sciences study design

All studies must disclose on these points even when the disclosure is negative.

Sample size	48 NPC1 variants were measured in this study. The severity score and age of first neurologic symptom of 27 anonymous patients were included in this study.
Data exclusions	No data is excluded.
Replication	The precise number of repeats (n) are indicated in the supplementary excel sheet for each experiment of each variant.
Randomization	N/A
Blinding	N/A

Reporting for specific materials, systems and methods

We require information from authors about some types of materials, experimental systems and methods used in many studies. Here, indicate whether each material, system or method listed is relevant to your study. If you are not sure if a list item applies to your research, read the appropriate section before selecting a response.

Materials & experimental systems

n/a	Involved in the study	
☐	☒	Antibodies
☐	☒	Eukaryotic cell lines
☒	☐	Palaeontology
☒	☐	Animals and other organisms
☒	☐	Human research participants
☒	☐	Clinical data

Methods

n/a	Involved in the study	
☒	☐	ChIP-seq
☒	☐	Flow cytometry
☒	☐	MRI-based neuroimaging

Antibodies

Antibodies used	All antibody information including clone numbers and company source are provided in the Methods section.
Validation	All antibodies were validated by the supplier for human samples, and were checked in the lab by Western Blotting on cell lysate and by comparing to the manufacturer's or in-house results.

Eukaryotic cell lines

Policy information about cell lines

Cell line source(s)	All the cell line sources are provided in the Methods section.
Authentication	The morphology of the cell lines is monitored through microscopic inspection. Stable silencing of endogenous NPC1 in all cell lines was validated by RT-PCR and Western blot analysis. The plasmids of all the variants were validated by sequencing as described in the Methods section. Transfected cell lines are monitored by Western blot analysis, RT-PCR and fluorescent measurements to confirm the level of expression and phenotype of the disease associated variants.
Mycoplasma contamination	No mycoplasma contamination was found in the cell lines used in this study.
Commonly misidentified lines (See ICLAC register)	No cell line listed by ICLAC was used.
